# Examining the Impact of Intrinsic Rewards on Employee Retention: Perceived Organizational Pride as a Mediator in Saudi Higher Education

**DOI:** 10.3390/bs16060982

**Published:** 2026-06-12

**Authors:** Hammad S. Alotaibi

**Affiliations:** College of Taraba, Taif University, Taif P.O. Box 11099, Saudi Arabia; hammad@tu.edu.sa

**Keywords:** task autonomy, personal growth and development opportunities, self-actualization, decision-making participation, perceived organizational pride, employee retention

## Abstract

This study examines the relationships between intrinsic motivation factors—task autonomy, personal growth and development opportunities, self-actualization, and decision-making participation—and employee retention, as well as the mediating role of perceived organizational pride. Using a quantitative cross-sectional survey, data were collected from 154 academic staff members at Taif University, Saudi Arabia. CFA supported the measurement model, and the hypotheses were tested using Hayes’ PROCESS macro. The findings show that all intrinsic motivation factors are positively associated with employee retention. Perceived organizational pride also mediates these relationships, suggesting that intrinsically motivating work conditions may support retention by strengthening employees’ pride in institutional membership. The results further indicate that developmental and participative factors show stronger associations with retention than task autonomy. This study contributes to employee retention research by integrating intrinsic motivation and identity-based explanations in the context of Saudi higher education. However, given the cross-sectional design and single-university sample, causal interpretation and generalizability should be treated with caution. The findings highlight the importance of growth-oriented, participative, and pride-enhancing work environments for supporting academic staff retention.

## 1. Introduction

Employee retention—an organization’s ability to keep its workforce, reducing voluntary turnover and associated hiring costs through strategies that foster engagement, satisfaction, and loyalty (Mitchell et al., 2001; Hom et al., 2017; Jiang et al., 2017)—has become a strategic priority in contemporary higher education, where institutional performance increasingly depends on intellectual capital, research continuity, and academic excellence (Bichsel et al., 2023; Tight, 2022). The departure of qualified faculty and administrative staff disrupts knowledge transfer, weakens program stability, and imposes substantial financial and reputational costs (O’Callaghan, 2024). In Saudi Arabia, this challenge is particularly salient as the higher education sector undergoes extensive transformation under Vision 2030, which seeks to elevate universities to global standards while simultaneously implementing structural, governance, and performance reforms (Mohiuddin et al., 2023; Abdullateef et al., 2023). Prior retention research has identified several factors that influence employees’ intention to remain, including salary, job satisfaction, organizational support, career opportunities, and work environment (Bichsel et al., 2023; Shahzad et al., 2024).

Although these studies provide useful insights, much of the literature remains focused on direct associations between work-related factors and retention. This limits understanding of the psychological processes through which employees decide to remain with their organizations. Intrinsic motivation factors are widely recognized as important for satisfying employees’ needs for autonomy, competence, and meaningful contribution (Deci & Ryan, 2000; Laguerre & Barnes-Farrell, 2025). *Intrinsic rewards*—such as task autonomy, opportunities for personal growth and development, self-actualization, and participation in decision-making—satisfy fundamental psychological needs for competence, autonomy, and meaningful contribution (Ryan & Deci, 2024; Tiffin et al., 2024). Within academic settings, where professional identity and intellectual fulfillment are central, these intrinsic factors may be particularly influential (Vela et al., 2023; Bichsel et al., 2023). However, while prior studies have acknowledged the motivational value of such factors, limited empirical attention has been given to the psychological mechanisms through which intrinsic motivation translates into employee retention, especially in non-Western institutional contexts (Shahzad et al., 2024; Schaefer et al., 2024).

Prior research on retention in Saudi higher education has largely emphasized structural and extrinsic factors, such as salary, job satisfaction, work environment, HR practices, and organizational support (Koshak et al., 2024; Altassan & Rahman, 2023). Yet retention remains a practical concern; for example, Koshak et al. (2024) reported that employee turnover in a public university in Jeddah became around 25% higher than its 2018 level. This creates a research tension: although intrinsic motivation and established mediators such as affective commitment, engagement, and organizational identification are known to explain retention (Meyer et al., 2012; Christian et al., 2011; Riketta, 2005), they may not fully capture the pride-based emotional pathway through which employees decide to remain.

Perceived organizational pride (POP) offers a theoretically appropriate explanatory mechanism because it reflects a self-conscious and evaluative emotion rooted in employees’ positive appraisal of their organization’s achievements, reputation, and external standing (Gouthier & Rhein, 2011; Kraemer & Gouthier, 2014; Schaefer et al., 2024; Schilpzand et al., 2025). Although several established constructs may explain employee retention, including affective commitment, job satisfaction, engagement, organizational identification, perceived organizational support, and institutional prestige, POP captures a distinct emotional pathway. Affective commitment reflects emotional attachment to the organization (Meyer & Allen, 1991), job satisfaction reflects employees’ evaluation of their job experiences, engagement captures energy and involvement in work roles (Kahn, 1990; Schaufeli et al., 2002), organizational identification reflects cognitive oneness with the organization (Ashforth & Mael, 1989), perceived organizational support reflects employees’ beliefs that the organization values and supports them (Eisenberger et al., 1986), and institutional prestige reflects external evaluations of organizational status (Smidts et al., 2001). By contrast, POP explains how employees convert their organization’s achievements, reputation, and social standing into personal self-worth and positive emotional attachment to membership. Thus, POP is not treated as a substitute for these established constructs but as a more specific affective–evaluative mechanism through which intrinsic motivation may translate into stronger retention intentions.

This distinction is particularly important in Saudi higher education. Saudi universities occupy a highly visible role in the national transformation agenda, especially under Vision 2030 and the Human Capability Development Program, where higher education is positioned as a core driver of knowledge development, national competitiveness, and human capability building (Mohiuddin et al., 2023; Vision 2030, 2021). In such a context, employees’ motivation to remain may not depend only on satisfaction with their job, perceived organizational support, or emotional attachment to the institution. It may also depend on whether they feel proud to belong to a university that is viewed as reputable, socially valuable, and aligned with national development priorities. This pathway is also consistent with Saudi Arabia’s collectivist cultural context, where group membership, loyalty, institutional reputation, and collective identity are socially meaningful (Hofstede, 2001; Tajfel & Turner, 1986; Triandis, 1995). Accordingly, POP provides additional explanatory value by showing how intrinsically motivated employees may develop a stronger desire to remain when their work allows them to feel personally associated with a respected and socially meaningful academic institution.

Despite growing recognition of retention challenges within Saudi universities, important gaps remain in the literature. First, existing research in the region has predominantly focused on extrinsic factors such as compensation, promotion, and contractual stability, with comparatively limited attention to intrinsic motivational drivers (Bichsel et al., 2023; Shahzad et al., 2024). Second, the role of POP as an explanatory mechanism between intrinsic motivation and retention has received limited empirical attention, particularly in collectivist cultural settings where identity-based and emotion-based processes may operate differently from those in Western contexts (Schaefer et al., 2024; Schilpzand et al., 2025). Third, the relative contributions of the intrinsic motivation dimensions—autonomy, development, self-actualization, and participation—have not been systematically examined within a unified framework in Saudi higher education (Mohiuddin et al., 2023).

**Figure 1:**
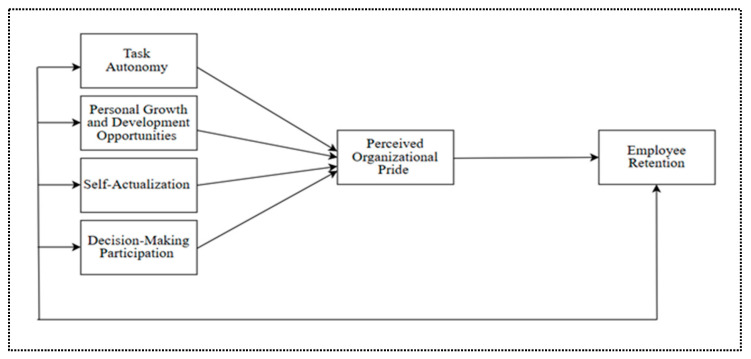
Theoretical model.

This study contributes by integrating intrinsic motivation theory with social identity theory to explain employee retention through an identity-based mechanism. While intrinsic motivation research has traditionally focused on performance, engagement, and satisfaction outcomes, its explanatory power for retention has been less systematically examined through mediating psychological constructs. By positioning perceived organizational pride as the key mechanism linking intrinsic motivational factors to retention, this study extends Self-Determination Theory beyond need satisfaction toward identity internalization processes. In doing so, it bridges motivational and identification-based perspectives, demonstrating that intrinsically fulfilling work environments do not merely enhance positive attitudes but also shape employees’ organizational self-concept. This integration advances retention theory by highlighting that employee retention is not solely driven by resource availability or job characteristics, but by the extent to which intrinsically motivating conditions cultivate pride and strengthen identity alignment with the institution. The study’s conceptual model is illustrated in Figure 1.

## 2. Literature Review and Hypotheses Development

### 2.1. Task Autonomy and Employee Retention

Task autonomy refers to the degree of discretion and independence employees have in scheduling their work and determining the procedures used to carry it out (Hackman & Oldham, 1980; Otoo, 2024). Within job characteristics theory, autonomy is considered a core motivational attribute because it strengthens employees’ experienced responsibility for work outcomes and enhances intrinsic motivation (Hackman & Oldham, 1980; Cotič et al., 2025). Employees who perceive greater autonomy are more likely to experience ownership of their work, greater psychological empowerment, and stronger motivation to continue contributing to the organization (O’Callaghan, 2024; Otoo, 2024; Tian et al., 2020).

Prior research suggests that retention may also be explained by compensation, promotion opportunities, job satisfaction, perceived organizational support, institutional prestige, affective commitment, and external labor market opportunities (Eisenberger et al., 1986; Meyer & Allen, 1991; Mitchell et al., 2001; Smidts et al., 2001). In addition, autonomy may not always lead to retention if employees experience excessive workload, unclear expectations, or insufficient organizational support. In such cases, autonomy may be interpreted less as empowerment and more as additional responsibility without adequate resources. Therefore, task autonomy should be understood as one important motivational condition among several possible explanations of employee retention.

From a self-determination theory perspective, autonomy is especially relevant because it satisfies employees’ basic psychological need for self-governance and volition (Deci & Ryan, 2000; Ryan & Deci, 2024). When employees feel that they have meaningful discretion over how they perform their work, they are more likely to internalize organizational goals and view continued membership as personally valuable (Gagné & Deci, 2005; Tiffin et al., 2024). Empirical studies have shown that autonomy is positively associated with work engagement, job satisfaction, and commitment, while being negatively related to withdrawal behaviors and turnover intentions (Ko & Oh, 2025; Otoo, 2024). In higher education, task autonomy may be particularly important because academic and professional employees often value discretion, independent judgment, and participation in decisions related to teaching, research, service, and administrative responsibilities (Bichsel et al., 2023; Vela et al., 2023). Thus, we argue that when universities provide employees with sufficient autonomy, employees may perceive the institution as a place where they can exercise professional agency, pursue meaningful work goals, and maintain a sense of personal control. These experiences can strengthen their willingness to remain with the institution.

**Hypothesis 1.**  *Task autonomy is positively related to employee retention.*

### 2.2. Personal Growth and Development Opportunities and Employee Retention

Personal growth and development opportunities refer to employees’ perceptions that their organization provides meaningful avenues for skill development, career advancement, and professional development (London, 1993; Shahzad et al., 2024). Prior research suggests that perceived developmental support is an important predictor of employee retention, as it signals that the organization values employees’ long-term contributions and future potential (Singh et al., 2023; Shahzad et al., 2024). Employees who perceive stronger opportunities for learning and advancement are more likely to develop affective commitment, career optimism, and a stronger intention to remain with the organization (Shahzad et al., 2024).

Nevertheless, development opportunities do not provide a complete explanation for employee retention. Employees may remain with an organization because of several other factors, including competitive compensation, job security, institutional prestige, supportive leadership, work–life balance, perceived organizational support, and limited external employment opportunities (Eisenberger et al., 1986; Meyer & Allen, 1991; Mitchell et al., 2001; Smidts et al., 2001). Moreover, development opportunities may not always translate into retention. For example, employees who gain new skills and credentials may become more attractive to external employers, which could increase mobility rather than strengthen retention. Similarly, if developmental support is perceived as symbolic, unequal, or disconnected from actual promotion opportunities, its positive effect on retention may be weakened. Thus, personal growth and development opportunities should be understood as an important but not exclusive explanation for why employees decide to remain with their organization.

From the perspective of self-determination theory, development opportunities are theoretically relevant because they fulfill the basic psychological need for competence, thereby strengthening intrinsic motivation and the internalization of organizational goals (Deci & Ryan, 2000; Ryan & Deci, 2024; Laguerre & Barnes-Farrell, 2025). When employees perceive that they are acquiring new skills, progressing professionally, and expanding their future career possibilities, they are more likely to experience their work as meaningful and personally valuable (Tiffin et al., 2024; Laguerre & Barnes-Farrell, 2025). This sense of competence can foster stronger attachment to the institution and reduce withdrawal cognitions.

In higher education contexts, personal growth and development opportunities may be particularly salient because professional identity is closely tied to scholarly development, research productivity, teaching expertise, and academic progression. Access to research funding, promotion pathways, training programs, conference participation, and structured mentoring can signal that the university supports employees’ long-term academic and professional success (Vela et al., 2023). In Saudi higher education, where universities are increasingly expected to contribute to national human capability development and institutional excellence, such opportunities may strengthen employees’ belief that remaining with the institution supports both personal advancement and broader academic contribution. Therefore, we propose the following hypothesis:

**Hypothesis 2.**  *Personal growth and development opportunities are positively related to employee retention.*

### 2.3. Self-Actualization and Employee Retention

Self-actualization refers to the realization of one’s full potential through meaningful, challenging, and personally fulfilling work experiences (Maslow, 1943; Ryan & Deci, 2024). Originating from humanistic psychology, self-actualization reflects individuals’ desire to grow, express their capabilities, and achieve purpose in their professional roles (Maslow, 1943; Ryff, 1989). In organizational contexts, employees who perceive their work as personally meaningful and aligned with their values may develop a stronger desire to remain because the organization becomes a setting through which they can pursue fulfillment, professional identity, and personal growth (Ryan & Deci, 2024; Schilpzand et al., 2025).

At the same time, self-actualization should not be viewed as the only explanation for employee retention. Employees may remain with an organization because of affective commitment, job satisfaction, perceived organizational support, organizational identification, job embeddedness, institutional prestige, or limited external career alternatives (Eisenberger et al., 1986; Meyer & Allen, 1991; Mitchell et al., 2001; Smidts et al., 2001). Moreover, meaningful and fulfilling work may not automatically lead to retention when employees face excessive workload, limited promotion opportunities, weak institutional support, or stronger career prospects elsewhere. Thus, self-actualization is best understood as a motivational pathway that complements, rather than replaces, established explanations of retention.

Drawing on self-determination theory, self-actualization is relevant because it reflects the satisfaction of employees’ autonomy and competence needs, both of which foster intrinsic motivation and the internalization of organizational goals (Deci & Ryan, 2000; Laguerre & Barnes-Farrell, 2025). When employees experience their work as a source of personal fulfillment, authentic self-expression, and professional accomplishment, they are more likely to perceive continued organizational membership as meaningful and valuable (Ryan & Deci, 2024; Tiffin et al., 2024). Empirical studies also show that meaningful work, a core element of self-actualization, is positively associated with affective commitment and negatively related to turnover intentions (Schilpzand et al., 2025).

In higher education, self-actualization may be especially relevant because academic work is closely linked to intellectual growth, scholarly contribution, teaching purpose, and professional identity. Faculty and academic staff often seek opportunities to contribute knowledge, mentor students, conduct research, and participate in institutional development. When universities provide conditions that allow employees to realize these aspirations, employees may be more likely to view the institution as a meaningful place to continue their careers (Bichsel et al., 2023; Vela et al., 2023). This is particularly relevant in Saudi higher education, where universities are increasingly expected to contribute to national development, knowledge creation, and human capability building. Therefore, we propose the following hypothesis:

**Hypothesis 3.**  *Self-actualization is positively related to employee retention.*

### 2.4. Decision-Making Participation and Employee Retention

Decision-making participation refers to the extent to which employees are involved in decisions that affect their work, professional roles, and the institution’s broader direction (Ogu, 2024; Kim & Cho, 2024). Participative practices can strengthen employees’ sense of influence, inclusion, and voice by signaling that their expertise and opinions are valued. Prior research suggests that employee participation is associated with higher job satisfaction, stronger organizational commitment, and lower turnover intentions because it allows employees to feel respected and involved in matters that shape their work environment (Kim & Cho, 2024; Ogu, 2024; Shahzad et al., 2024).

However, participation in decision-making is not the only explanation for employee retention. Employees may remain in an organization because of affective commitment, job satisfaction, perceived organizational support, institutional prestige, job security, compensation, career development opportunities, or limited external alternatives (Eisenberger et al., 1986; Meyer & Allen, 1991; Mitchell et al., 2001; Smidts et al., 2001). Moreover, participation may not always strengthen retention if employees perceive decision-making processes as symbolic, selective, or lacking real influence. In such cases, participation may create frustration rather than attachment, particularly when employees are invited to contribute ideas but see little evidence that their input affects institutional decisions. Therefore, participation in decision-making should be viewed as an important relational and motivational condition, rather than as a complete explanation of retention.

Drawing on self-determination theory, decision-making participation is theoretically relevant because it satisfies employees’ psychological need for autonomy by allowing them to experience volition, influence, and self-direction in organizational affairs (Deci & Ryan, 2000; Ryan & Deci, 2024). Participation may also support competence and relatedness needs because employees can contribute their expertise, interact with institutional leaders, and feel recognized as meaningful members of the organization (Tiffin et al., 2024; Laguerre & Barnes-Farrell, 2025). These experiences can strengthen intrinsic motivation, psychological ownership, and organizational attachment, thereby reducing employees’ intentions to leave (Kim & Cho, 2024; Shahzad et al., 2024).

In higher education institutions, participation in decision-making is particularly relevant because shared governance, academic voice, and professional consultation are central to academic work. Faculty and academic staff often expect involvement in decisions related to curriculum development, research policy, quality assurance, student affairs, and institutional strategy. When universities provide meaningful opportunities for such participation, employees may feel respected, trusted, and recognized as contributors to institutional development (Vela et al., 2023; Ogu, 2024). In Saudi higher education, where universities are undergoing reform and transformation under national development agendas, participation may further strengthen employees’ sense that they are active contributors to institutional progress rather than passive recipients of organizational change. Therefore, we propose the following hypothesis:

**Hypothesis 4.**  *Decision-Making Participation is positively related to employee retention.*

### 2.5. Perceived Organizational Pride as a Mediator

Perceived organizational pride reflects employees’ positive emotional attachment to their institution’s achievements and reputation (Schaefer et al., 2024; Schilpzand et al., 2025). Task autonomy enhances employees’ sense of responsibility and professional respect, which strengthens their psychological attachment to the organization (Cotič et al., 2025; O’Callaghan, 2024). When employees perceive that their institution trusts them with discretion and independence, they are more likely to evaluate their affiliation positively and experience pride. Pride, in turn, reinforces affective commitment and intention to remain (Schaefer et al., 2024; Schilpzand et al., 2025). Development opportunities signal institutional investment in employees’ long-term success (Shahzad et al., 2024; Singh et al., 2023). Such investment enhances positive organizational evaluation and collective self-esteem. Empirical evidence shows that career development strengthens organizational identification and commitment (Singh et al., 2023). Consequently, employees who perceive growth opportunities are more likely to feel proud of their institution, thereby enhancing retention intentions. Self-actualization through meaningful and fulfilling work fosters strong alignment between personal and organizational values (Ryan & Deci, 2024; Schilpzand et al., 2025). When institutions enable employees to realize their potential, they cultivate pride in membership, which enhances emotional attachment and retention (Schaefer et al., 2024; Schilpzand et al., 2025). Participation enhances inclusion and voice, strengthening identification and collective esteem (Kim & Cho, 2024). When employees feel influential in institutional decisions, they are more likely to develop pride, which, in turn, increases their intention to remain (Schaefer et al., 2024; Kim & Cho, 2024).

Self-determination theory posits that fulfilling the psychological needs for autonomy, competence, and relatedness enhances intrinsic motivation and the internalization of organizational values (Ryan & Deci, 2024; Laguerre & Barnes-Farrell, 2025). Task autonomy directly satisfies the need for autonomy; personal growth and development opportunities strengthen competence; self-actualization reinforces both autonomy and competence through meaningful contribution; and decision-making participation fosters autonomy and relatedness by enhancing voice and inclusion (Tiffin et al., 2024; Laguerre & Barnes-Farrell, 2025). When these psychological needs are satisfied, employees develop a stronger identification with their organization. Social identity theory further explains that individuals derive part of their self-concept from valued group membership (Tajfel & Turner, 1986; Schaefer et al., 2024). Intrinsically motivating environments, therefore, not only enhance internal motivation but also cultivate collective self-esteem in the form of organizational pride. This pride strengthens affective attachment and reduces withdrawal intentions, ultimately leading to higher employee retention (Schilpzand et al., 2025; Schaefer et al., 2024). Thus, organizational pride functions as the identity-based mechanism through which intrinsic motivational conditions translate into sustained organizational membership. Thus, we propose the following hypotheses:

**Hypothesis 5.**  *Perceived organizational pride mediates the relationship between task autonomy and employee retention.*

**Hypothesis 6.**  *Perceived organizational pride mediates the relationship between personal growth and development opportunities and employee retention.*

**Hypothesis 7.**  *Perceived organizational pride mediates the relationship between self-actualization and employee retention.*

**Hypothesis 8.**  *Perceived organizational pride mediates the relationship between decision-making participation and employee retention.*

## 3. Methods

### 3.1. Sampling and Procedure

This study employed a quantitative cross-sectional survey design using a self-administered questionnaire and convenience sampling. The research was conducted at Taif University (TU), Saudi Arabia, with a final sample of 154 academic staff members. To assess sample adequacy, we conducted a power analysis using G*Power 3.1. The analysis was based on a linear multiple regression model with a medium effect size (f^2^ = 0.15), alpha level of 0.05, statistical power of 0.80, and five predictors, reflecting the most complex regression equation in the proposed mediation model. The results indicated that a minimum sample size of 92 participants was required. The final sample size of 154 exceeded this threshold, suggesting that the study had adequate statistical power to test the proposed regression-based mediation model.

Convenience sampling was selected for its practicality in accessing a defined institutional population within a limited timeframe and for its frequent use in organizational behavior research in higher education contexts (Etikan et al., 2016; Sakkara & Bougie, 2016). Data collection followed a formal institutional protocol. An official request was submitted to the Deanship of Scientific Research at Taif University to obtain ethical approval.

After approval was granted, a formal communication was sent to the Human Resources (HR) department to facilitate academic staff’s access. The HR department assisted in distributing the survey link electronically across departments, ensuring broad coverage within the university. Participation was voluntary, and respondents were assured of confidentiality and anonymity to reduce social desirability bias. For data analysis, a two-step approach was adopted. First, confirmatory factor analysis (CFA) was conducted using AMOS to assess the measurement model, including construct reliability and validity. Second, the hypothesized direct and indirect effects were tested using Hayes’ PROCESS macro in SPSS v. 25, which is widely used for mediation analysis and is appropriate for small-to-medium sample sizes (Hair et al., 2019; Hayes, 2022). Bootstrapping procedures were applied to estimate confidence intervals for indirect effects, thereby enhancing the robustness of mediation testing.

### 3.2. Measures

The survey was administered in English, as it is commonly used in higher-education academic work and research communication in the study context. All constructs were measured using validated multi-item scales with responses recorded on a five-point Likert scale ranging from 1 (strongly disagree) to 5 (strongly agree). Although the survey was administered in English, we assessed the measures’ suitability for the Saudi higher education context. The items were reviewed for clarity, contextual relevance, and cultural appropriateness before data collection. Particular attention was given to constructs such as autonomy, decision-making participation, self-actualization, and perceived organizational pride, as these concepts may carry different meanings across cultural and institutional contexts. The review confirmed that the items were understandable for university employees and suitable for use in an English-administered survey in Saudi higher education. Task autonomy was assessed using four items adapted from Mottaz (1981). Personal growth and development were measured with five items from Presbitero et al. (2016). Self-actualization was captured using six items from Jones and Crandall (1986). Decision-making participation was assessed with six items from Arnold et al. (2000). Perceived organizational pride was measured using seven items adapted from Gouthier and Rhein (2011). Finally, Employee Retention was assessed using four items adapted from Kyndt et al. (2009).

## 4. Results

### 4.1. Descriptive Statistics

Table 1 reports means, standard deviations, and intercorrelations among the study variables. All focal constructs were positively and significantly correlated (*p* < 0.01), providing preliminary support for the hypothesized relationships. Reliability coefficients (diagonal values) ranged from 0.83 to 0.94, indicating satisfactory internal consistency.

### 4.2. Common Method Bias

Harman’s single-factor test was conducted to assess the potential influence of common method bias. All retained measurement items were entered into an unrotated exploratory factor analysis using principal component analysis. The results showed that the first factor explained 29.23% of the total variance, which is below the commonly used 50% threshold. This suggests that common method bias is unlikely to be a serious concern in the present study.

### 4.3. Model Fitness

Table 2 presents the results of the confirmatory factor analysis. During measurement validation, three items were removed: TA3, SA1, and PGD5. These items showed weak standardized factor loadings (0.427, 0.469, and 0.359, respectively), falling below the recommended 0.50 threshold for item retention (Hair et al., 2019; Kline, 2016). Because low-loading items may weaken convergent validity and measurement quality (Bagozzi & Yi, 1988; Fornell & Larcker, 1981), their removal was considered appropriate. However, item deletion was not based solely on statistical criteria. Consistent with recommendations that scale refinement should also preserve content validity and construct domain coverage (Haynes et al., 1995; Netemeyer et al., 2003), the retained items were reviewed conceptually. The remaining items continued to capture the core meanings of task autonomy, self-actualization motivation, and personal growth and development motivation. After removing these items, all retained indicators loaded significantly on their respective constructs, with factor loadings ranging from 0.656 to 0.980. Composite reliability values ranged from 0.79 to 0.98, exceeding the recommended 0.70 threshold, while average variance extracted values ranged from 0.56 to 0.91, surpassing the 0.50 criterion. The six-factor measurement model also demonstrated acceptable fit to the data: χ^2^(363) = 703.19, χ^2^/df = 1.94, CFI = 0.93, TLI = 0.93, IFI = 0.93, and RMSEA = 0.07. Overall, these results provide evidence of convergent validity, internal consistency reliability, and acceptable measurement model fit.

### 4.4. Discriminant Validity

Table 3 reports the Fornell–Larcker test of discriminant validity. The square root of AVE for each construct (diagonal values) exceeds its correlations with other constructs (off-diagonal values). For example, TA (0.75), SA (0.84), PGD (0.89), DMP (0.88), POP (0.90), and ER (0.95) are all greater than their highest inter-construct correlations. Although some correlations are relatively high (e.g., POP–ER = 0.82), they remain below the corresponding √AVE values. Discriminant validity was further assessed using the heterotrait–monotrait ratio of correlations (HTMT). The HTMT values ranged from 0.403 to 0.832, and all values were below the conservative 0.85 threshold, providing additional evidence of discriminant validity. Although the association between perceived organizational pride and employee retention was relatively strong, this relationship is conceptually understandable, as employees who feel proud of their organization may be more inclined to remain with it. However, the two constructs are theoretically distinct. Perceived organizational pride reflects an affective–evaluative response to the organization’s achievements, reputation, and social standing. In contrast, employee retention reflects employees’ intention or willingness to remain members of the organization. Thus, pride captures an emotional appraisal of organizational membership, while retention reflects a behavioral intention to continue employment. Empirically, the corresponding HTMT value was 0.819, which remains below the recommended threshold. These findings indicate that perceived organizational pride and employee retention are closely related but empirically distinct constructs. Therefore, discriminant validity is supported.

### 4.5. Hypotheses Results

**Figure 2:**
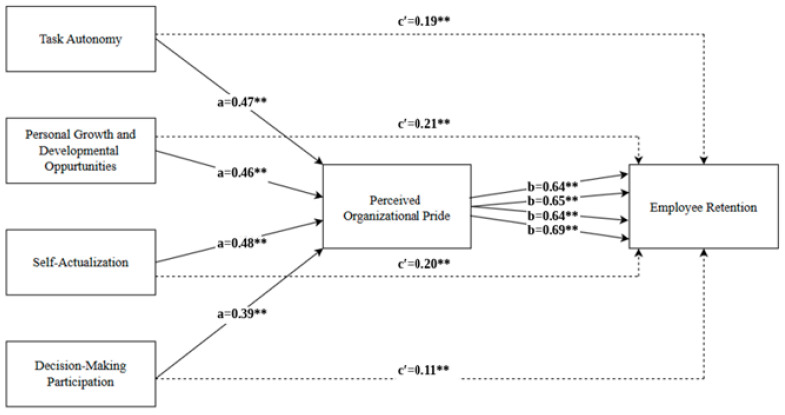
Solid arrows represent indirect path components (a and b paths). Dashed arrows represent direct effects (c’). ** *p* < 0.01.

Table 4 and Figure 2 present the results of the hypothesis testing. H1 predicted that task autonomy is positively related to employee retention. The results supported this hypothesis, showing a significant positive relationship between task autonomy and employee retention (B = 0.19 **, 95% CI [0.08, 0.30]). H2 predicted that personal growth and development opportunities are positively associated with employee retention, and the results supported this hypothesis (B = 0.21 **, 95% CI [0.10, 0.31]). H3 predicted that self-actualization is positively associated with employee retention (B = 0.20 **, 95% CI [0.10, 0.31]). H4 predicted that decision-making participation is positively associated with employee retention, and the results provided support for this relationship (B = 0.11 **, 95% CI [0.01, 0.22]). Therefore, H1–H4 were supported.

H5 predicted that perceived organizational pride mediates the relationship between task autonomy and employee retention. The indirect effect was significant (B = 0.30 **, 95% CI [0.20, 0.40]), supporting H5. H6 predicted that perceived organizational pride mediates the relationship between personal growth and development opportunities and employee retention. This indirect effect was also significant (B = 0.29 **, 95% CI [0.19, 0.42]), supporting H6. H7 predicted that perceived organizational pride mediates the relationship between self-actualization and employee retention, and the results confirmed this mediation effect (B = 0.31 **, 95% CI [0.21, 0.41]), supporting H7. Finally, H8 predicted that perceived organizational pride mediates the relationship between decision-making participation and employee retention, which was also supported (B = 0.27 **, 95% CI [0.16, 0.38]). Thus, H5–H8 were supported.

## 5. Discussion

This study provides new evidence on the role of intrinsic motivation factors in strengthening employee retention by examining how employees respond to task autonomy, personal growth and development opportunities, self-actualization, and decision-making participation. Drawing on intrinsic motivation and social identity perspectives, our study revealed that these motivational factors enhance employee retention both directly and indirectly through perceived organizational pride. Specifically, employees are more likely to remain with their organization when they experience greater autonomy in their work, receive opportunities for growth and development, perceive their work as a means of self-actualization, and are allowed to participate in decision-making processes.

These findings are consistent with previous research that autonomy and competence development strengthen employee motivation, commitment, and positive work attitudes, all of which reduce turnover intentions (Chandra et al., 2025). Employee participation in decision-making has likewise been shown to increase organizational belonging and retention (Teodorovicz et al., 2024). Moreover, enabling self-actualization—allowing employees to work at their full trained capacity—has been identified as a key mechanism for lowering turnover (Guan et al., 2025). Taken together, prior studies demonstrate that autonomy, development opportunities, meaningful work, and participation increase employees’ psychological attachment to the organization and reduce their likelihood of leaving.

More importantly, this study extends the employee retention literature by identifying perceived organizational pride as a key psychological mechanism. The findings suggest that intrinsic motivation factors do not merely improve retention by making work more satisfying; rather, they help employees develop pride in being associated with their organization. This pride strengthens their emotional connection with the organization and encourages them to remain. Recent empirical work supports this explanatory pathway. Organizational pride has been shown to explain how high-performance HR practices contribute to innovative work behavior (Deepa et al., 2025). Similarly, Brown et al. (2022) found that institutional reputation reduces turnover intentions by strengthening employees’ pride in their organization. Recent evidence also suggests that pride in organizational membership can lower turnover intention by reducing the negative consequences of misfit between employees’ needs and service quality (Yongho Hyun et al., 2024). In this way, our study responds to calls for a deeper understanding of the psychological processes through which motivational work conditions influence employee retention. By positioning perceived organizational pride as an important explanatory pathway, this study expands the theoretical scope of retention research and highlights the importance of creating work environments that support autonomy, growth, fulfillment, and employee participation.

### 5.1. Theoretical Implications

This study makes several important contributions to the literature on employee retention and intrinsic motivation. First, it advances retention research by integrating multiple intrinsic motivation factors—task autonomy, personal growth and development opportunities, self-actualization, and decision-making participation—within a single explanatory framework. Although prior research has examined these variables independently (Hackman & Oldham, 1980; Deci & Ryan, 2000; O’Callaghan, 2024), limited work has assessed their relative and simultaneous effects on retention. By modeling these intrinsic motivational drivers together, the study provides a more comprehensive and comparative understanding of how internally driven psychological resources shape employees’ decisions to remain with their organization.

Second, the findings extend intrinsic motivation and work design theories by demonstrating that higher-order developmental and self-fulfillment factors may exert stronger influences on retention than structural job characteristics alone (Cotič et al., 2025; Laguerre & Barnes-Farrell, 2025). While autonomy has long been recognized as a core motivational element, the stronger effects of growth opportunities, participation, and self-actualization suggest that employees increasingly value meaningful development and psychological fulfillment (Bichsel et al., 2023; Shahzad et al., 2024). This refines existing motivational frameworks by highlighting the evolving importance of developmental intrinsic drivers in contemporary organizational contexts.

Third, this study contributes by identifying perceived organizational pride as a central psychological mechanism linking intrinsic motivation to employee retention. Although motivational theories explain how fulfilling work enhances positive attitudes, empirical research has rarely positioned organizational pride as the mediating pathway through which intrinsic motivation translates into retention outcomes (Schaefer et al., 2024; Schilpzand et al., 2025). By integrating motivational theory with social-determination perspectives, the study demonstrates that intrinsically motivating conditions foster pride, thereby strengthening employees’ emotional attachment and desire to remain. Fourth, the study shifts the retention literature beyond transactional or economic explanations by emphasizing identity-based and affective processes. The results suggest that employees do not remain solely because of job features or external incentives; rather, they stay when intrinsically motivating environments cultivate pride and psychological alignment with the organization. This offers a more psychologically grounded and identity-informed explanation of retention.

### 5.2. Practical Implications

The findings offer several implications for managerial practice, particularly within Saudi Arabian higher education institutions. First, universities should recognize that intrinsic motivation plays an important role in retaining academic and administrative staff. While compensation and contractual stability remain important, the results suggest that retention efforts should also focus on autonomy, developmental opportunities, meaningful contribution, and participation in decision-making. Rather than relying only on salary-based or contractual retention strategies, university leaders should design retention policies that address employees’ psychological attachment to their institution. In the context of Saudi Arabia’s higher education reforms under Vision 2030, creating intrinsically motivating academic environments may help universities sustain academic talent during institutional transformation.

Second, university leadership should prioritize structured professional development pathways. The strong effects of personal growth and development opportunities indicate that faculty members are more likely to remain when they perceive clear opportunities for research advancement, international collaboration, promotion, and skill enhancement. Saudi universities can strengthen retention by investing in research funding, conference support, sabbatical programs, and transparent promotion systems that signal long-term institutional commitment. More specifically, universities could introduce annual individual development plans, mentoring schemes for early-career academics, internal research grants, support for international publication, and clearly communicated promotion criteria. These practices would make developmental support visible and credible rather than symbolic.

Third, at the departmental level, heads of departments and program leaders can introduce mentoring arrangements, encourage collegial support for research and publication, involve faculty members in curriculum- and program-related decisions, and create informal opportunities for academic staff to participate in departmental planning. Such initiatives are relatively feasible because they depend mainly on local leadership practices and departmental culture. By contrast, recommendations such as competitive research grants, formal publication support schemes, transparent promotion criteria, workload policies, and institution-wide participative governance mechanisms require broader institutional or policy-level support. These initiatives depend on university leadership, human resource policies, budget allocation, and alignment with national higher education priorities. Therefore, improving employee retention in Saudi higher education requires both local departmental actions that strengthen day-to-day academic experiences and wider institutional reforms that create fair, supportive, and development-oriented employment systems.

Fourth, fostering self-actualization in academic roles is essential. Faculty members in higher education often seek intellectual fulfillment, autonomy in teaching and research, and opportunities to contribute meaningfully to knowledge creation. Administrators should therefore reduce unnecessary bureaucratic constraints and allow greater flexibility in curriculum design, research agendas, and pedagogical innovation. For example, departments could give academics more discretion in selecting research themes, designing course content, leading academic initiatives, and participating in interdisciplinary projects. Supporting academic freedom and intellectual growth can enhance intrinsic motivation and strengthen employees’ willingness to remain.

Fifth, the results highlight the importance of participative governance structures. Decision-making participation contributes to retention, both directly and indirectly, through perceived organizational pride. In Saudi Arabian universities, where governance structures may be relatively hierarchical, introducing inclusive committees, faculty councils, and transparent communication channels can enhance employees’ sense of voice and influence. Universities could involve academic staff in curriculum reform, research strategy, accreditation planning, departmental budgeting, and quality-assurance decisions. When faculty members feel that their perspectives are taken into account in institutional decisions, they may develop greater pride and attachment to the university.

Most importantly, the study underscores the strategic value of cultivating perceived organizational pride. Universities should actively build a strong institutional identity by celebrating research achievements, international rankings, accreditation milestones, and societal contributions. Public recognition of faculty accomplishments, internal award systems, and visible leadership support can enhance collective pride. However, pride-building efforts should be authentic and linked to employees’ actual contributions. For instance, universities can regularly communicate how faculty research, teaching, community engagement, and student mentoring contribute to institutional reputation and national development goals. In this way, organizational pride becomes more than institutional branding; it becomes a mechanism through which employees see their work as meaningful and socially valued.

Overall, Saudi Arabian higher education institutions seeking sustainable talent retention should move beyond generic retention practices and develop integrated retention systems that combine professional development, academic voice, meaningful work design, and pride-enhancing recognition practices. Such practices may be especially useful for strengthening academic staff retention in institutions undergoing reform, globalization, and performance-based transformation.

### 5.3. Limitation and Future Direction

Despite its contributions, this study has several limitations. First, its cross-sectional design limits causal inference because all variables were measured at one point in time. Although the proposed relationships are theoretically supported, the design does not allow us to establish strict temporal ordering among intrinsic motivation, perceived organizational pride, and employee retention. Therefore, the mediation results should be interpreted as evidence of indirect associations rather than definitive causal effects. Future research should use longitudinal, time-lagged, cross-lagged, or experimental designs to examine how intrinsic motivation and perceived organizational pride develop over time and subsequently influence employee retention.

Second, the study used convenience sampling from a single institution, Taif University in Saudi Arabia, with a relatively limited sample size of 154 respondents. This sampling approach may restrict the external validity of the findings and increase the possibility of selection bias, as participants may not fully represent employees across Saudi higher education institutions. Institutional culture, governance systems, employment policies, and labor market conditions may differ across universities and national contexts. Although the sample size was adequate for the analyses conducted, future studies should justify sample adequacy through formal power analysis and replicate the model using larger, more diverse samples across multiple public and private universities and in different cultural settings to assess broader generalizability and contextual boundaries.

Third, the reliance on self-reported data from a single source may raise concerns about common-method bias. To minimize this concern, Harman’s single-factor test was conducted to assess the potential presence of common method variance, but future studies should use stronger procedures, such as marker-variable techniques or latent common method factor models.

Fourth, this study focused on perceived organizational pride as the key explanatory mechanism. Future research could examine whether organizational pride explains additional variance in retention beyond established mechanisms such as psychological empowerment, job crafting, and thriving at work. Such comparative testing would help clarify the incremental explanatory value of perceived organizational pride in employee retention research.

Finally, future studies could investigate boundary conditions that may shape the strength of the proposed relationships. Context-specific factors such as organizational culture, tenure, academic rank, and generational differences may influence when intrinsic motivation is more likely to translate into organizational pride and retention. Examining these moderators would further refine the theoretical model and enhance its practical relevance for higher education institutions.

### 5.4. Conclusions

This study suggests that intrinsic motivation factors—task autonomy, personal growth and development opportunities, self-actualization, and decision-making participation—are positively associated with employee retention in the context of Saudi higher education. The findings also indicate that perceived organizational pride mediates the proposed relationships. In particular, the results suggest that developmental and participative aspects of work are important retention-related factors, as employees may be more willing to remain when they perceive opportunities for growth, meaningful contribution, and involvement in institutional matters. By integrating intrinsic motivation with social identity perspectives, this study provides a psychologically grounded explanation of employee retention in higher education. However, given the cross-sectional design, convenience sample from a single university, modest sample size, and reliance on self-reported data, the findings should be interpreted with caution. Future research should validate the proposed model using longitudinal, multi-source, and multi-institutional designs.

## Figures and Tables

**Table 1:** Means, standard deviations, and correlations.

Variable	M	SD	1	2	3	4	5	6	7	8
1. Gender	1.36	0.48								
2. Age	36.1	0.68	−0.08							
3. TA	3.03	0.74	0.01	−0.15	**0.** **83**					
4. SA	2.15	0.83	0.01	−0.41	0.23 **	**0.** **88**				
5. PGD	2.63	0.97	0.04	−0.37	0.27 **	0.15 **	**0** **.87**			
6. DMP	2.98	1.03	0.02	−0.38	0.23 **	0.16 **	0.18 **	**0** **.94**		
7. POP	2.36	1.02	−0.04	−0.36	0.48 **	0.48 **	0.45 **	0.39 **	**0.** **89**	
8. ER	2.47	1.16	−0.02	−0.30	0.52 **	0.52 **	0.49 **	0.40 **	0.76 **	**0** **.91**

Note(s). N = 154. ** Correlation is significant at the 0.01 level (2-tailed). TA—Task Autonomy; SA—Self-Actualization; PGD—Personal Growth and Development Opportunities; DMP—Decision-Making Participation; POP—Perceived Organizational Pride; ER—Employee Retention. Bold Values shown at diagonal are alpha reliability values.

**Table 2:** Confirmatory factor analysis.

Construct	Item	Factor Loading	CR	AVE
TA	TA1	0.681	0.79	0.56
	TA2	0.723		
	TA3	0.427 (Drop)		
	TA4	0.838		
SA	SA1	0.469 (Drop)		
	SA2	0.787	0.91	0.71
	SA3	0.656		
	SA4	0.819		
	SA5	0.834		
	SA6	0.895		
PGD	PGD1	0.891	0.94	0.80
	PGD2	0.909		
	PGD3	0.909		
	PGD4	0.864		
	PGD5	0.359 (Drop)		
DMP	DMP1	0.882	0.95	0.78
	DMP2	0.951		
	DMP3	0.860		
	DMP4	0.882		
	DMP5	0.801		
	DMP6	0.827		
POP	POP1	0.939	0.97	0.81
	POP2	0.954		
	POP3	0.880		
	POP4	0.820		
	POP5	0.942		
	POP6	0.800		
	POP7	0.932		
ER	ER1	0.980	0.98	0.91
	ER2	0.979		
	ER3	0.946		
	ER4	0.900		

**Table 3:** Discriminant validity (Fornell–Larcker criterion).

Construct	TA	SA	PGD	DMP	POP	ER
TA	**0.75**					
SA	0.70	**0.84**				
PGD	0.56	0.84	**0.89**			
DMP	0.48	0.69	0.71	**0.88**		
POP	0.48	0.77	0.74	0.75	**0.90**	
ER	0.38	0.64	0.63	0.61	0.82	**0.95**

**Table 4:** Hypothesis results.

Hypotheses	Relationship	B	95% CI
H1	Task Autonomy → Employee Retention	0.19 **	[0.08 0.30]
H2	Personal Growth and Development Opportunities → Employee Retention	0.21 **	[0.10, 0.31]
H3	Self-Actualization → Employee Retention	0.20 **	[0.10, 0.31]
H4	Decision-Making Participation → Employee Retention	0.11 **	[0.01, 0.22]
H5	Task Autonomy → Perceived Organizational Pride → Employee Retention	0.30 **	[0.20, 0.40]
H6	Personal Growth and Development Opportunities → Perceived Organizational Pride→ Employee Retention	0.29 **	[0.19, 0.42]
H7	Self-Actualization → Perceived Organizational Pride→ Employee Retention	0.31 **	[0.21, 0.41]
H8	Decision-Making Participation → Perceived Organizational Pride→ Employee Retention	0.27 **	[0.16, 0.38]

Note(s). N = 154. ** *p* < 0.01.

## Data Availability

All the data are included in the article.
